# Exploring Depression and Nutritional Covariates Amongst US Adults using Shapely Additive Explanations

**DOI:** 10.1002/hsr2.1635

**Published:** 2023-10-20

**Authors:** Alexander A. Huang, Samuel Y. Huang

**Affiliations:** ^1^ Northwestern University Feinberg School of Medicine Chicago Illinois USA; ^2^ Virginia Commonwealth University School of Medicine Richmond Virginia USA

**Keywords:** machine learning, NHANES, nutrition, PHQ‐9, XGBoost

## Abstract

**Background:**

Depression affects personal and public well‐being and identification of natural therapeutics such as nutrition is necessary to help alleviate this public health concern.

**Objective:**

The study aimed to identify feature importance in a machine learning model using solely nutrition covariates.

**Methods:**

A retrospective analysis was conducted using a modern, nationally representative cohort, the National Health and Nutrition Examination Surveys (NHANES 2017−2020). Depressive symptoms were evaluated using the validated 9‐item Patient Health Questionnaire (PHQ‐9), and all adult patients (total of 7929 individuals) who completed the PHQ‐9 and total nutritional intake questionnaire were included in the study. Univariable regression was used to identify significant nutritional covariates to be included in a machine learning model and feature importance was reported. The acquisition and analysis of the data were authorized by the National Center for Health Statistics Ethics Review Board.

**Results:**

7929 patients met the inclusion criteria in this study. The machine learning model had 24 out of a total of 60 features that were found to be significant on univariate analysis (*p* < 0.01 used). In the XGBoost model the model had an Area Under the Receiver Operator Characteristic Curve (AUROC) = 0.603, Sensitivity = 0.943, Specificity = 0.163. The top four highest ranked features by gain, a measure of the percentage contribution of the covariate to the overall model prediction, were Potassium Intake (Gain = 6.8%), Vitamin E Intake (Gain = 5.7%), Number of Foods and Beverages Reported (Gain = 5.7%), and Vitamin K Intake (Gain 5.6%).

**Conclusion:**

Machine learning models with feature importance can be utilized to identify nutritional covariates for further study in patients with clinical symptoms of depression.

## 1 INTRODUCTION

Depression is a pressing global health concern, affecting over 300 million people of all ages and backgrounds.^1^, ^2^, ^3^ According to the World Health Organization, depression is one of the leading causes of disability worldwide, and its prevalence continues to rise.^4^, ^5^ Its impact on individuals, communities, and economies is significant, with reduced productivity and increased healthcare costs.^1^, ^6^, ^7^ There is a growing recognition of the importance of addressing depression as a public health priority, and efforts are being made at both national and international levels to identify risk factors and promote effective treatments.^8^, ^9^, ^10^ As the stigma around mental health decreases and more people seek help for depression, access to coverage for depression care through insurance is also increasing.^11^, ^12^, ^13^ With rising levels of psychological stress due to occupation and other factors, effective strategies are urgently needed to prevent and treat depression.

Given the detrimental effects of depression, it is imperative to increase research on potential treatments and interventions.^14^, ^15^ Lifestyle changes, including diet and exercise, have been identified as potential avenues for treatment.^16^, ^17^, ^18^, ^19^, ^20^ In addition, there is a growing body of evidence on specific nutritional targets that are inversely associated with depression. In our enhanced data set, we examine all the nutrition covariates to determine their potential impact on depressive symptoms.^21^, ^22^

Although depression has been recognized as a significant contributor to increasing mortality and morbidity, our understanding of specific nutritional risk factors that are strongly associated with depressive symptoms is limited. To address this gap in the literature, we will utilize transparent machine learning methods such as Shapely Additive Explanations (SHAP), model explanations, and model gain statistics to identify the most important features for clinical depressive symptoms. This study will leverage data from the National Health and Nutrition Examination Surveys (NHANES) 2017−2020 cohort, a large and nationally representative sample of US adults, to investigate the potential impact of nutrition on depressive symptoms. By using these advanced analytical techniques, we hope to gain a deeper understanding of the nutritional factors that contribute to depressive symptoms and inform future interventions to improve mental health outcomes.

## 2 METHODS

Individuals who completed the demographic, dietary, exercise, and mental health questionnaire and had laboratory and physical exam data were the subjects of a retrospective, cross‐sectional cohort study using the publicly available NHANES. The National Center for Health Statistics (NCHS) Ethics Review Board granted permission for the study's data collection and analysis. Before data analysis in this retrospective cohort, all data—medical records, survey information, and demographic data—were completely anonymized, and all individuals consented to their data being made public.

### 2.1 Data set and cohort selection

The NCHS developed the National Health and Nutrition Examination Survey (NHANES 2017−2020), which has been utilized to evaluate the health and nutritional status of the population of the United States. The Centers for Disease Control and Prevention (CDC) carried out a series of complex, cross‐sectional, multi‐stage surveys on a nationally representative cohort of the population of the United States to collect data on health, nutrition, and physical activity for the NHANES data set. We looked at adult (less than 18 years old) patients in the NHANES data set who had completed the demographic, dietary, exercise, and mental health questionnaires and had data from their physical and laboratory examinations.

### 2.2 Assessment of symptoms of clinical depression

The validated 9‐item Patient Health Questionnaire (PHQ‐9) depression scale was used to measure depressive symptoms. PHQ‐9 demonstrates strong agreement with interviews conducted by inpatient psychiatrists when it asks for symptoms of depression over the past 2 weeks. Questions include: “Over the last 2 weeks, how often have you been bothered by any of the following problems: (1) Little or no pleasure in doing things? (2) Feeling down, depressed, or hopeless? (3) Trouble falling or staying asleep, or sleeping too much? (4) Feeling tired or having little energy? (5) Poor appetite or overeating? (6) Feeling bad about yourself—or that you are a failure or have let yourself or your family down? (7) Trouble concentrating on things, such as reading the newspaper or watching television? (8) Moving or speaking so slowly that other people could have noticed? Or so fidgety or restless that you have been moving a lot more than usual? (9) Thoughts that you would be better off dead, or thoughts of hurting yourself in some way?” and were assessed on a scale from 0 to 3: 0 = Not at all, 1 = Several Days, 2 = more than half the days, and 3 = nearly every day. According to literature a threshold score of ≥10 was considered to have clinical depression with a sensitivity of 89% and a specificity of 89% when compared to a psychiatrists diagnosis.^23^, ^24^, ^25^

### 2.3 Independent variable

In NHANES, the dietary questionnaire contained the potential model covariates. The NHANES data set yielded a total of 60 nutrition covariates. PHQ‐9 scores were combined with all covariates.

### 2.4 Model construction and statistical analysis

The PHQ‐9 cut‐off value of greater than or equal to 10 was used as the outcome in univariate logistic models to find nutritional covariates associated with clinical depression. The final machine‐learning model included covariates with a *p* Value of 0.01 on univariate analysis. To ensure that all of the 60 covariates used in the machine learning models were strong independent covariates, an initial filter was performed using univariable logistic models on the data set. Additionally, physicians were able to examine whether risk factors were clinically relevant thanks to this initial filtering. Model importance statistics from machine‐learning models were used to identify pertinent risk factors following initial filtering.

Due to its increased predictive accuracy in healthcare prediction and its prevalence in the literature, the machine learning model XGBoost was utilized. XGBoost was chosen due to other studies using the NHANES cohort that found it to be the most efficacious—providing the best combination of training efficiency, model accuracy, and transparency.^26^, ^27^, ^28^ The final set of model fit parameters were calculated using an approach that involved dividing the data into training and testing sets in an 80:20 ratio. The area under the receiver operator characteristic curve (AUROC), sensitivity, specificity, positive predictive value, negative predictive value, prevalence, detection rate, detection prevalence, and balanced accuracy were the model fit parameters used in this study.

### 2.5 Model feature importance statistics and SHAP visualization

To find risk factors having clinically relevant depression, model covariates were ranked by Gain, Cover, and Frequency. The feature's relative contribution to the model is called the Gain. The Cover is the total number of observations that were made about this feature. The percentage of times a feature appears in the machine‐learning model's trees is known as the Frequency. Due to its ease of interpretation, the Gain statistic was chosen as the method for ranking features according to importance: the proportion of the final prediction that was influenced by the covariate. SHAP explanations were utilized to visualize the continuous covariates with the strongest relationship between the potential risk factors and a sleep disorder.

## 3 RESULTS

Table 1 shows of the 7929 patients that met the inclusion criteria in this study, the mean number of foods and beverages reported was 14.64 (SD = 5.87). Mean potassium intake was 2,497.92 mg (SD = 1,274.54), mean alpha‐tocopherol intake was 8.91 mg (SD = 5.96), mean Vitamin K intake was 71.93 mcg (SD = 68.73), and mean phosphorous intake was 103.85 mg (SD = 94.46).

**Table 1:** Demographic variables.

Total	Total	PHQ‐9 ≥ 10	PHQ‐9 < 10	*p* Value
	7929	550	7379	
Number of foods and beverages reported	14.64 (5.87)	12.77 (5.59)	14.78 (5.87)	*p* < 0.01
Grams of protein	73.97 (34.59)	68.14 (34.43)	74.41 (34.56)	*p* < 0.01
Grams of fiber	15.32 (8.93)	13.29 (8.58)	15.47 (8.94)	*p* < 0.01
Vitamin B6 (mg)	1.93 (1.53)	1.73 (1.42)	1.95 (1.54)	*p* < 0.01
Folate (mcg)	337.36 (189.77)	311.99 (192.39)	339.27 (189.45)	*p* < 0.01
Total choline (mg)	193.35 (115.08)	171.45 (112.45)	195.00 (115.11)	*p* < 0.01
Added Vitamin B12 (mcg)	309.16 (168.90)	280.97 (165.16)	311.29 (169.00)	*p* < 0.01
Vitamin K (mcg)	71.93 (68.73)	58.89 (62.60)	72.91 (69.08)	*p* < 0.01
Phosphorus (mg)	103.85 (94.46)	86.90 (88.09)	105.13 (94.80)	*p* < 0.01
Zinc (mg)	268.61 (124.73)	240.51 (120.49)	270.73 (124.80)	*p* < 0.01
Sodium (mg)	9.51 (5.10)	8.78 (5.04)	9.56 (5.10)	*p* < 0.01
Caffeine (mg)	2400.68 (1214.56)	2201.33 (1340.34)	2415.68 (1203.34)	*p* < 0.01
Theobromine (mg)	106.51 (61.20)	98.69 (59.78)	107.10 (61.27)	*p* < 0.01
Low fat and low cholesterol diet	15.31 (6.01)	13.72 (6.10)	15.43 (5.99)	*p* < 0.01
Dietary fiber (gm)	16.17 (10.50)	13.70 (9.13)	16.35 (10.57)	*p* < 0.01
Vitamin E as alpha tocopherol (mg)	8.91 (5.96)	8.13 (5.90)	8.97 (5.96)	*p* < 0.01
Lutein zeaxanthin (mcg)	1173.37 (1292.12)	960.86 (1152.74)	1189.21 (1300.59)	*p* < 0.01
Total folate (mcg)	347.58 (185.64)	314.70 (187.40)	350.03 (185.29)	*p* < 0.01
Food folate (mcg)	198.99 (114.72)	176.00 (110.96)	200.71 (114.81)	*p* < 0.01
Folate DFE (mcg)	448.99 (254.51)	410.13 (259.38)	451.89 (253.92)	*p* < 0.01
Magnesium (mg)	282.66 (128.65)	258.10 (129.29)	284.49 (128.43)	*p* < 0.01
Iron (mg)	13.12 (6.79)	12.26 (7.01)	13.18 (6.77)	*p* < 0.01
Copper (mg)	1.11 (0.55)	1.01 (0.55)	1.12 (0.55)	*p* < 0.01
Potassium (mg)	2497.92 (1274.54)	2299.38 (1334.61)	2512.71 (1268.80)	*p* < 0.01

*Note*: Descriptive statistics for demographic characteristics and all covariates within the machine learning model, stratified by whether patients had clinically depressive symptoms as defined by PHQ‐9 greater than or equal to 10.

**Figure 1:**
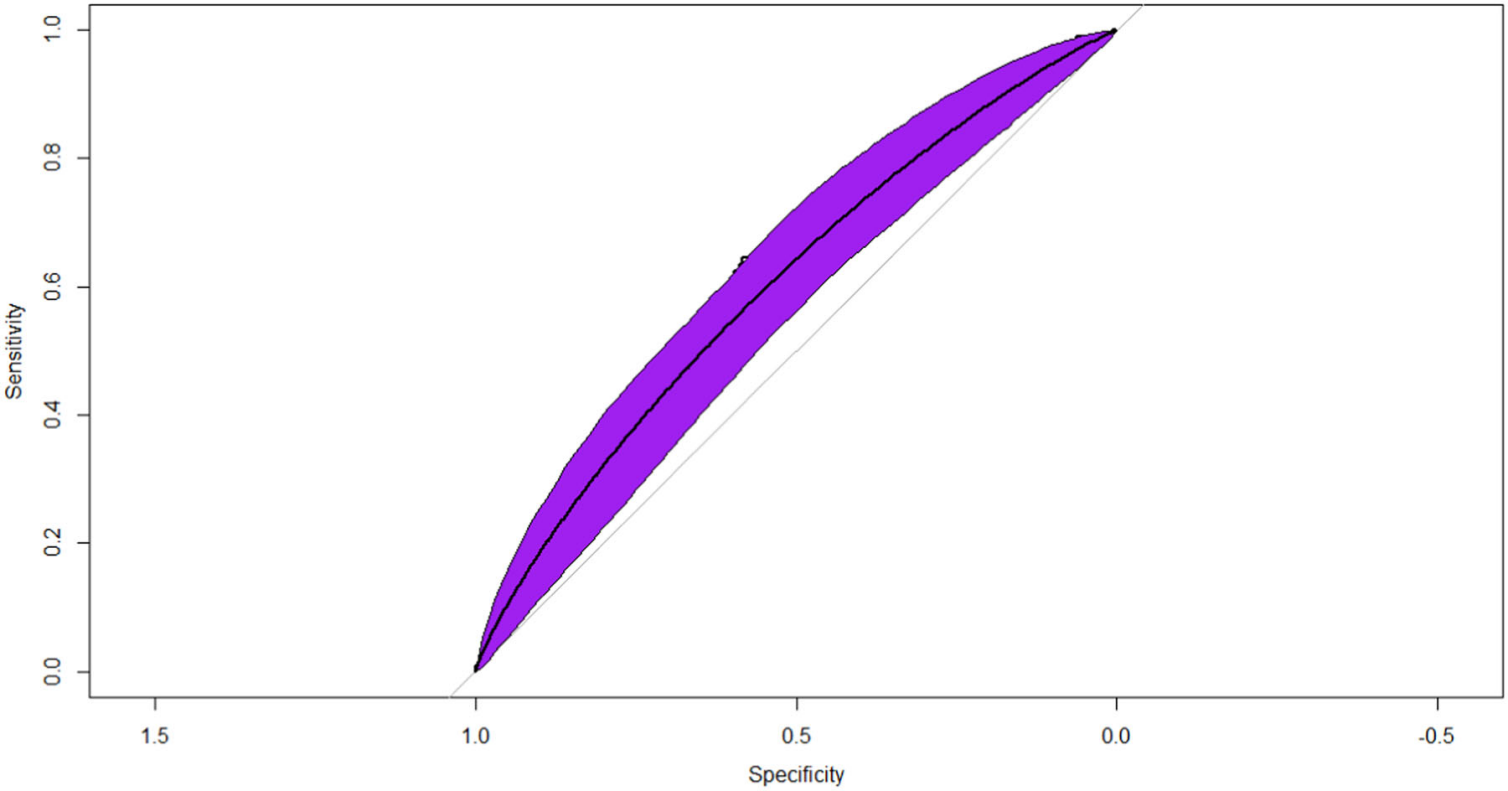
Receiver operator characteristic curve and model statistics. The receiver operating characteristic curve for the machine‐learning model predicting clinically depressive symptoms as evidenced as PHQ‐9 greater than or equal to 10. AUROC = 0.603 (*p* < 0.001). AUROC, Area Under the Receiver Operator Characteristic Curve.

The machine learning model had 30 out of a total 60 features that were found to be significant on univariate analysis (*p* < 0.01 used). These were fitted into the XGBoost model and Figure 1 shows an AUROC = 0.603, Sensitivity = 0.943, Specificity = 0.163 were observed. Tables 2 and 3 shows that the top four highest ranked features by gain, a measure of the percentage contribution of the covariate to the overall model prediction, were Potassium Intake (Gain = 6.8%), Vitamin E Intake (Gain = 5.7%), Number of Foods and Beverages Reported (Gain = 5.7%), and Vitamin K Intake (Gain 5.6%).

**Table 2:** Model gain statistics.

Feature	Gain	Cover	Frequency
Potassium (mg)	0.068	0.078	0.055
Vitamin E as alpha tocopherol (mg)	0.057	0.046	0.056
Number of foods and beverages reported	0.057	0.058	0.078
Vitamin K (mcg)	0.056	0.045	0.050
Phosphorus (mg)	0.050	0.026	0.043
Grams of fiber	0.048	0.026	0.050
Grams of protein	0.048	0.032	0.054
Lutein zeaxanthin (mcg)	0.047	0.042	0.048
Folate (mcg)	0.047	0.049	0.045
Low fat and low cholesterol diet	0.044	0.067	0.038
Magnesium (mg)	0.044	0.046	0.042
Dietary fiber (gm)	0.043	0.063	0.046
Vitamin B6 (mg)	0.043	0.047	0.044
Total choline (mg)	0.041	0.065	0.041
Caffeine (mg)	0.041	0.062	0.042
Food folate (mcg)	0.034	0.022	0.030
Zinc (mg)	0.032	0.021	0.032
Copper (mg)	0.031	0.029	0.031
Iron (mg)	0.031	0.023	0.030
Total folate (mcg)	0.031	0.026	0.032
Sodium (mg)	0.030	0.030	0.030
Added Vitamin B12 (mcg)	0.028	0.037	0.031
Theobromine (mg)	0.027	0.036	0.028
Folate DFE (mcg)	0.024	0.023	0.022

*Note*: The Gain, Cover, and Frequency of all covariates within the XGBoost model. The Gain represents the relative contribution of the feature to the model and is the most important metric of model importance within this study. Covariates ordered according to the Gain statistic.

**Table 3:** Model metrics.

Sensitivity	0.943
Specificity	0.163
Positive predictive value	0.972
Negative predictive value	0.083
Prevalence	0.969
Detection rate	0.914
Detection prevalence	0.94
Balanced accuracy	0.553

**Figure 2:**
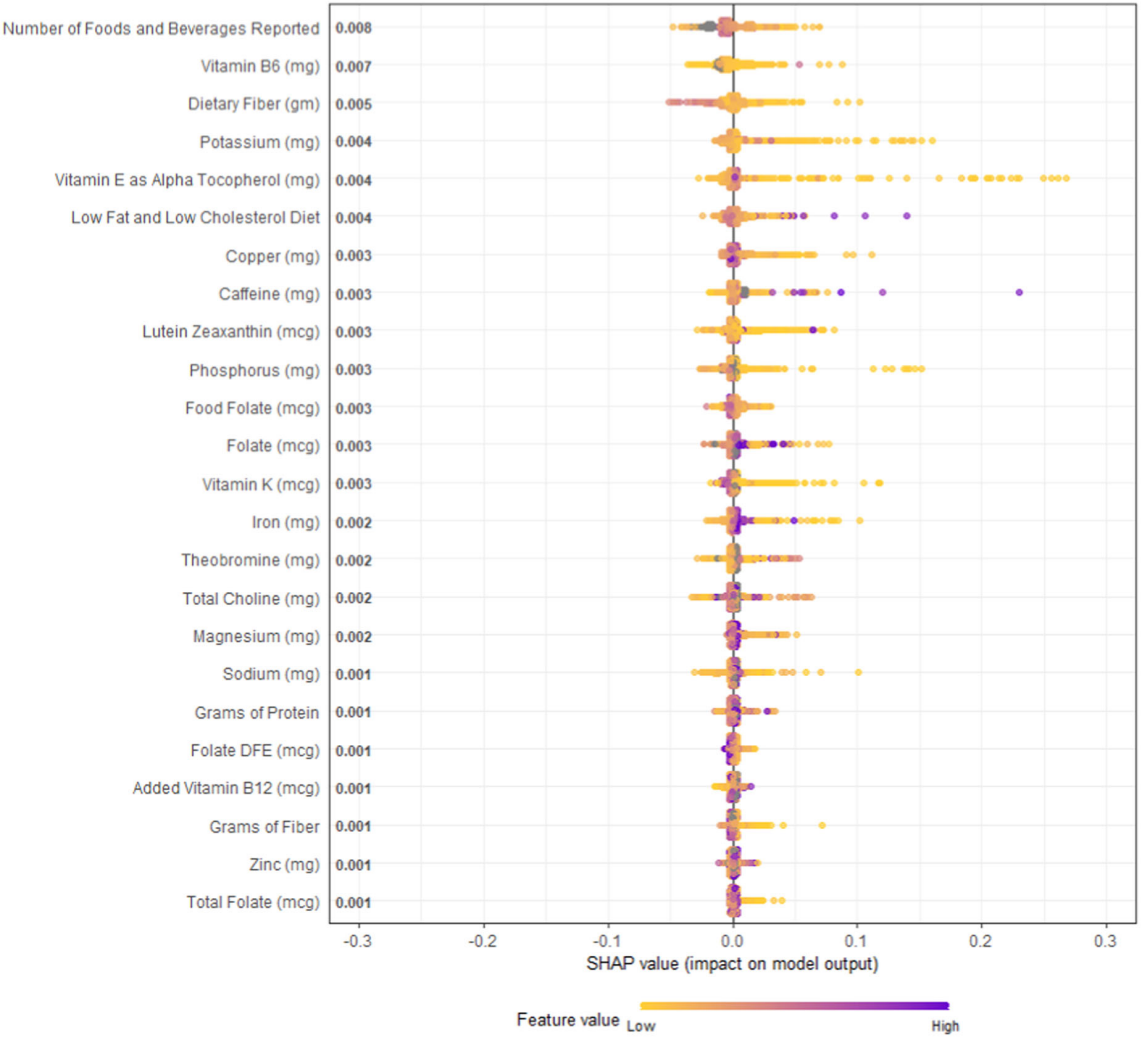
Overall SHAP explanations. SHAP explanations, purple color representing higher values of the covariate while yellow representing lower values of the covariate. X‐axis is the change in log‐odds for clinically relevant depression. SHAP, Shapely Additive Explanations.

In Figure 2, overall SHAP explanations can be seen for all the statistically significant nutrition covariates on univariable regression.

**Figure 3:**
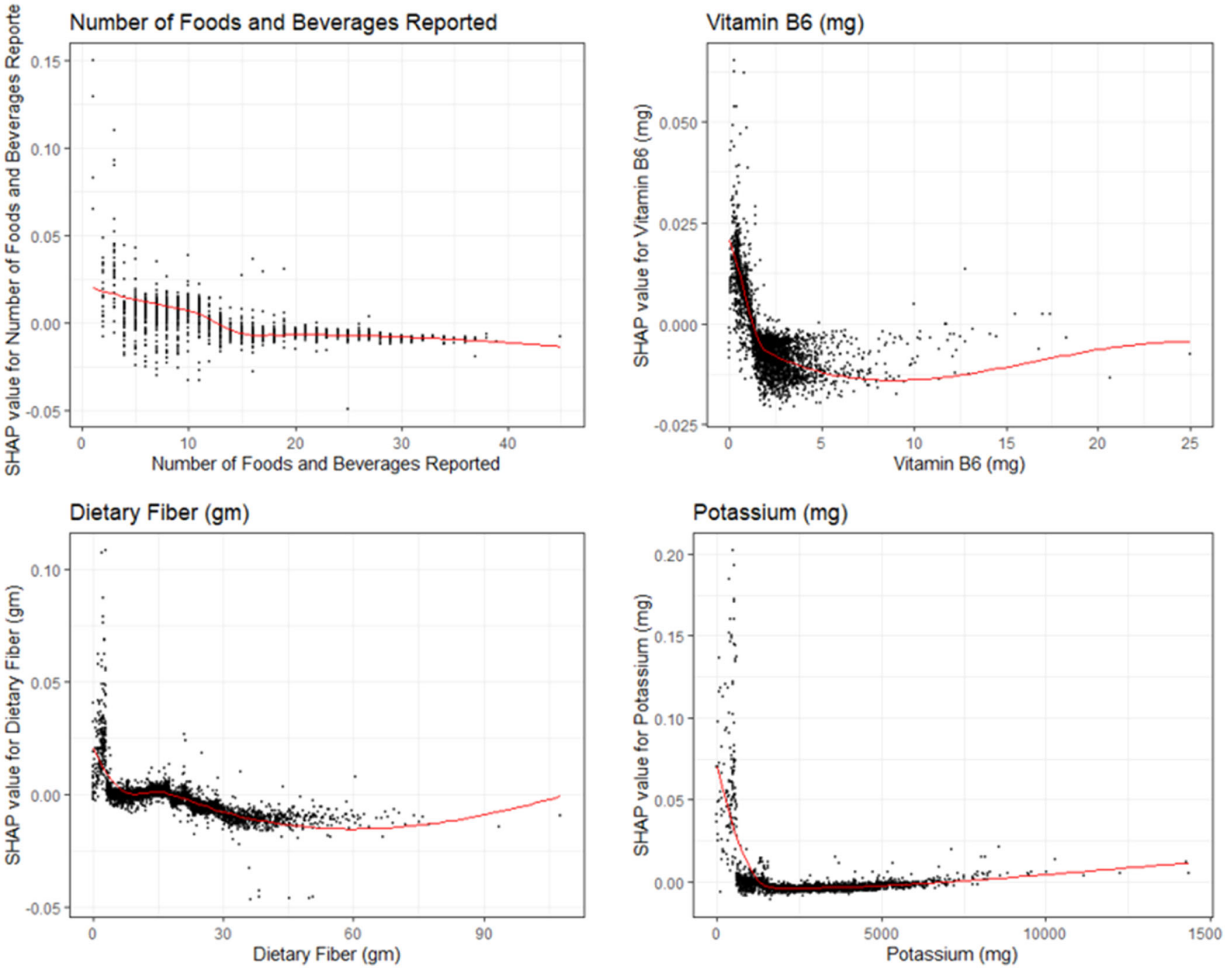
SHAP explanations for the top 4 continuous covariates sorted by gain statistics. SHAP explanations, covariate value on the x‐axis, change in log‐odds on the y‐axis, red line represents the relationship between the covariate and log‐odds for CAD, each black dot represents an observation. Covariates: top left—PHQ‐9, top right—Body weight, bottom left—patient age, bottom right—waist circumference. PHQ‐9, 9‐item Patient Health Questionnaire; SHAP, Shapely Additive Explanations.

In Figure 3 SHAP visualizations were conducted for the top four continuous covariates by model cover. We observed that increased number of foods and beverages reported were strongly associated with decreased odds of clinically depressive symptoms. Vitamin B6 intake had a curvilinear relationship with increasing Vitamin B6 intake up to 1.3 mg per day associated with decreased odds of PHQ‐9 greater than or equal to 10. Similarly, fiber and potassium had similar effects with increasing fiber up to 5 g and potassium up to 2300 mg associated with decreased odds of clinically depressive symptoms. The remainder of the SHAP visualizations can be seen in the Supporting Informatio Materials (S1−S20).

## 4 DISCUSSION

In this retrospective, cross sectional cohort of United States adults, a machine learning model utilizing only nutritional covariates had great sensitivity and positive predictive value for depression (AUROC = 0.603, Sensitivity = 0.943, Specificity = 0.163). The greatest predictors for clinically relevant depression included Potassium intake, Vitamin E intake, number of food and beverages consumed, and Vitamin K intake.

Machine learning and artificial intelligence models have been effectively used to predict mental health disorders.^29^, ^30^, ^31^ Prior studies have accurately predicted the presence of depression or depression relapse using machine‐learning methods from a variety of datasets that take into account physical parameters such as weight and blood pressure to bloodwork using numerous machine‐learning methods.^29^, ^30^, ^32^, ^33^ These studies highlight the utility of machine learning models in identifying patients at risk for depression. What our study adds to the literature is a large data set (*N* = 7929) that contains targeted information on nutritional intake of individuals.

Many studies have also been pushing for increased transparency in modeling in general, but especially so in the field of psychiatry where syndrome and disorders are already so difficult to understand by themselves.^34^, ^35^, ^36^, ^37^ Our study utilizes a novel paradigm recently described in literature that combines the commonly used XGBoost algorithm with SHAP to describe how the model is predicting each feature and how important it is.

The visualizations completed for the top four continuous covariates were concordant with current literature: there is strong epidemiological evidence that good diet is associated with decreased risk of depression.^22^ Many authors have noted statistically significant associations between Vitamin B6, Dietary Fiber, Potassium Intake, Vitamin E, Low Fat and Low Cholesterol Diet, Copper, Caffeine, Lutein Zeaxanthin, Phosphorous, Food Folate, Folate, Vitamin K, Iron, Theobromine, Total Choline, Magnesium, Sodium, Grams of Protein, Vitamin B12, Fiber, and Zinc similarly to our study.^38^, ^39^, ^40^, ^41^, ^42^, ^43^ Since visualizations for risk factors match literature relationships, we have increased confidence that the machine learning model is able to capture the actual physiological relationships of these covariates.^44^, ^45^, ^46^, ^47^, ^48^, ^49^, ^50^, ^51^ These transparent machine‐learning tools allow for increased confidence that these algorithms are picking up true signal within these covariates to predict the presence of depression rather than just replicating potential biases stemming from systemic data‐quality errors that are present within the data set. Additionally, these SHAP visualizations allow us to interpret that the increase predictive power of these machine‐learning methods is associated with the ability for these nonparametric methods to more accurately capture the nonlinear interactive relationship between the covariates, rather than just over‐fitting the model to get increased accuracy.^52^, ^53^

The greatest strength of this algorithmic method for identification of the covariates is the ability to search through hundreds of covariates systematically without relying upon judgment form the researcher, which may be muddled by potential personal biases. This method also allows for the ranking of the relative importance of each of these covariates through the cover statistic, which allows us to obtain the relative contribution to the prediction each covariate has and thus infer from there an estimate for the relative contribution to true risk for depression that each patient has. Another strength is that after these covariates are selected and the model built, SHAP visualizations can be used to make sure that each of the covariate either matches current literature understandings of the covariate's association with a depression or in the case of a discrepancy, allow researchers to validate the plausibility of this feature and then evaluate for potential errors in data‐quality. One issue with the model is the poor balanced accuracy and specificity. There may be a high number of false positives generated from the data set because of a variety of factors including the heterogeneity of the population. If the population is law and heterogeneity it will make it more difficult for the algorithm to identify meaningful patterns and relationships thus explaining the for accuracy and low specificity.

### 4.1 Limitations

A potential weakness to this machine‐learning analysis was the retrospective nature of this cohort, leading to potential bias. However, this was limited by the use of training and testing sets to be able to minimize the errors that come with overfitting. Furthermore, visualizations of SHAP allow researchers to test for physiologic plausibility of each of these covariates and allows for effective analysis by researchers of whether these effects are due to true signal or if they are just noise that may be contributing to a type‐1 error. Given the analysis of the strengths and weaknesses of these methods, we argue that use of machine‐learning methods can be an effective first step in the identification of risk‐factors that can then be further selected by clinicians based upon the specific clinical presentation.

## 5 CONCLUSION

Machine learning models can be utilized to predict depression using only nutritional information.

## AUTHOR CONTRIBUTIONS

**Alexander A. Huang**: Conceptualization; investigation; methodology; visualization; writing—original draft; writing—review and editing. **Samuel Y. Huang**: Conceptualization; data curation; formal analysis; investigation; resources; writing—original draft; writing—review and editing. All authors have read and approved the final version of the manuscript. Feature Importance can show the contribution of nutritional covariates and their impact on Depression.

## CONFLICT OF INTEREST STATEMENT

The authors declare no conflict of interest.

## TRANSPARENCY STATEMENT

The lead author Samuel Y. Huang affirms that this manuscript is an honest, accurate, and transparent account of the study being reported; that no important aspects of the study have been omitted; and that any discrepancies from the study as planned (and, if relevant, registered) have been explained.

## Supporting information

Supporting information.

## Data Availability

The data from this cohort can be found on the NHANES section of the CDC website. Data described in the manuscript are present at: https://wwwn.cdc.gov/nchs/nhanes/continuousnhanes/default.aspx?cycle=2017-2020. Code book and analytic code will be made available upon request to huangs8@vcu.edu. The Corresponding Author Samuel Y. Huang had full access to all of the data in this study and takes complete responsibility for the integrity of the data and the accuracy of the data analysis.

## References

[1] Chodavadia P , Teo I , Poremski D , Fung DSS , Finkelstein EA . Prevalence and economic burden of depression and anxiety symptoms among Singaporean adults: results from a 2022 web panel. BMC Psychiatry. 2023;23(1):104. 36782116 10.1186/s12888-023-04581-7 PMC9925363

[2] Othmani A , Zeghina AO , Muzammel M . A model of normality inspired deep learning framework for depression relapse prediction using audiovisual data. Comput Meth Programs Biomed. 2022;226:107132. 10.1016/j.cmpb.2022.107132 36183638

[3] Swainson J , Reeson M , Malik U , Stefanuk I , Cummins M , Sivapalan S . Diet and depression: a systematic review of whole dietary interventions as treatment in patients with depression. J Affect Disord. 2023;327:270‐278. 36738997 10.1016/j.jad.2023.01.094

[4] Higgins C , Sharma S , Bimali I , et al. Cross‐sectional study examining the epidemiology of chronic pain in Nepal. PAIN Reports. 2023;8(2):e1067. 36818647 10.1097/PR9.0000000000001067 PMC9928837

[5] López Jaramillo AM , Rangel Gómez MG , Morales Chainé S , López Montoya A , Lira Chávez IA , Cruz‐Piñeiro R . Mental, neurological and substance use disorders among the Latino migrant population in the United States who visited the Health Windows and mobile health units in 2021. Front Public Health. 2023;11:959535. 36815159 10.3389/fpubh.2023.959535 PMC9939505

[6] Haider MB , Basida B , Kaur J . Major depressive disorders in patients with inflammatory bowel disease and rheumatoid arthritis. World J Clin Cases. 2023;11(4):764‐779. 36818627 10.12998/wjcc.v11.i4.764 PMC9928699

[7] Li TH , Kamin L , George J , Saiz FS , Meyer P . Impact of the COVID‐19 pandemic on treatment for mental health needs: a perspective on service use patterns and expenditures from commercial medical claims data. BMC Health Serv Res. 2023;23(1):163. 36797739 10.1186/s12913-023-09080-9 PMC9932413

[8] Thompson J , Marijam A , Mitrani‐Gold FS , Wright J , Joshi AV . Activity impairment, health‐related quality of life, productivity, and self‐reported resource use and associated costs of uncomplicated urinary tract infection among women in the United States. PLoS One. 2023;18(2):e0277728. 36724152 10.1371/journal.pone.0277728 PMC9891499

[9] Yan Y , Tu Y . The impact of China's urban and rural economic revitalization on the utilization of mental health inpatient services. Front Public Health. 2023;10:1043666. 36711421 10.3389/fpubh.2022.1043666 PMC9877533

[10] Yuan W . Identifying the effect of digital healthcare products in metaverse on mental health: studying the interaction of cyberchondria and technophobia. Am J Health Behav. 2022;46(6):729‐739. 36721275 10.5993/AJHB.46.6.15

[11] Feinstein JA , Bruckner AL , Chastek B , Anderson A , Roman J . Clinical characteristics, healthcare use, and annual costs among patients with dystrophic epidermolysis bullosa. Orphanet J Rare Dis. 2022;17(1):367. 36175960 10.1186/s13023-022-02509-0 PMC9524120

[12] Jaber DJ , Basheer HA , Albsoul‐Younes AM , et al. Prevalence and predictive factors for infertility‐related stress among infertile couples: a cross‐sectional study from Jordan and the occupied Palestinian territories. Saudi Med J. 2022;43(10):1149‐1156. 36261208 10.15537/smj.2022.43.10.20220411 PMC9994507

[13] Sulley S , Ndanga M , Saka AK . Prevalence of cannabis use and factors related to hospitalizations in the United States: a population‐based study using national inpatient sample between 2012 and 2018. Cureus. 2022;14(8):e28361. 36168355 10.7759/cureus.28361 PMC9507936

[14] Busch RM , Dalton JE , Jehi L , et al. Association of neighborhood deprivation with cognitive and mood outcomes in adults with pharmacoresistant temporal lobe epilepsy. Neurology. 2023;100:e2350‐e2359. 37076308 10.1212/WNL.0000000000207266 PMC10256132

[15] Carrasco‐Querol N , Gonzalez Serra G , Bueno Hernandez N , et al. Effectiveness and health benefits of a nutritional, chronobiological and physical exercise primary care intervention in fibromyalgia and chronic fatigue syndrome: SYNCHRONIZE+ mixed‐methods study protocol. Medicine (Baltimore). 2023;102(17):e33637. 37115043 10.1097/MD.0000000000033637 PMC10145802

[16] Bhargav H , Eiman N , Jasti N , et al. Composition of yoga‐philosophy based mental traits (Gunas) in major psychiatric disorders: a trans‐diagnostic approach. Front Psychol. 2023;14:1075060. 36818072 10.3389/fpsyg.2023.1075060 PMC9930472

[17] Liu Y , Wang H , Bai B , et al. Trends in unhealthy lifestyle factors among adults with stroke in the United States between 1999 and 2018. J Clin Med. 2023;12(3):1223. 36769871 10.3390/jcm12031223 PMC9917618

[18] Oakes‐Cornellissen A , Morton D , Rankin P , Renfrew M . Efficacy of a multimodal lifestyle intervention (The Lift Project) for improving the mental health of individuals with an affective mood disorder living in South Africa. Front Psychol. 2023;14:1127068. 36760459 10.3389/fpsyg.2023.1127068 PMC9905116

[19] Sekhri S , Verma A . Study of depression and its associated factors among patients of diabetes mellitus (DM) and hypertension (HTN) attending a primary health center (PHC) in a rural area of New Delhi, India. Cureus. 2023;15(1):e33826. 36819386 10.7759/cureus.33826 PMC9930692

[20] Teelucksingh S , Murali Govind R , Dobson R , Nelson‐Piercy C , Ovadia C . Treating vestibular migraine when pregnant and postpartum: progress, challenges and innovations. Int J Women's Health. 2023;15:321‐338. 36814528 10.2147/IJWH.S371491 PMC9940493

[21] Lu J , Yang J , Liang J , Mischoulon D , Nyer M . The descriptive analysis of depressive symptoms and white blood cell (WBC) count between the sexual minorities and heterosexual identifying individuals in a nationally representative sample: 2005‐2014. BMC Public Health. 2023;23(1):294. 36759803 10.1186/s12889-022-14847-6 PMC9909981

[22] Zhang L , Zhou Q , Shao LH , Hu XQ , Wen J , Xia J . Association of metabolic syndrome with depression in US adults: a nationwide cross‐sectional study using propensity score‐based analysis. Front Public Health. 2023;11:1081854. 36817886 10.3389/fpubh.2023.1081854 PMC9929360

[23] Kroenke K , Spitzer RL , Williams JBW . The PHQ‐9: validity of a brief depression severity measure. J Gen Intern Med. 2001;16(9):606‐613. 11556941 10.1046/j.1525-1497.2001.016009606.x PMC1495268

[24] Levis B , Benedetti A , Thombs BD . Accuracy of patient health questionnaire‐9 (PHQ‐9) for screening to detect major depression: individual participant data meta‐analysis. BMJ. 2019;365:l1476. 30967483 10.1136/bmj.l1476 PMC6454318

[25] Maurer DM , Raymond TJ , Davis BN . Depression: screening and diagnosis. Am Fam Physician. 2018;98(8):508‐515. 30277728

[26] Alleman K , Knecht E , Huang J , Zhang L , Lam S , DeCuypere M . Multimodal deep Learning‐Based prognostication in glioma patients: a systematic review. Cancers. 2023;15(2):545. 36672494 10.3390/cancers15020545 PMC9856816

[27] Alzahrani A , Alshehri M , AlGhamdi R , Sharma SK . Improved wireless medical cyber‐physical system (IWMCPS) based on machine learning. Healthcare. 2023;11(3):384. 36766959 10.3390/healthcare11030384 PMC9913988

[28] Iqbal JD , Christen M . The use of artificial intelligence applications in Medicine and the standard required for healthcare provider‐patient briefings‐an exploratory study. DIGITAL HEALTH. 2022;8:205520762211474. 10.1177/20552076221147423 PMC9806394 36601281

[29] Cho K , Choi J , Han S . Validation of depression determinants in caregivers of dementia patients with machine learning algorithms and statistical model. Front Med. 2023;10:1095385. 10.3389/fmed.2023.1095385 PMC9932916 36817793

[30] Milne‐Ives M , Selby E , Inkster B , Lam C , Meinert E . Artificial intelligence and machine learning in mobile apps for mental health: a scoping review. PLOS Digital Health. 2022;1(8):e0000079. 36812623 10.1371/journal.pdig.0000079 PMC9931284

[31] Ueda M , Watanabe K , Sueki H . Emotional distress during COVID‐19 by mental health conditions and economic vulnerability: retrospective analysis of survey‐linked twitter data with a semi‐supervised machine learning algorithm. J Med Internet Res. 2023;25:e44965. 36809798 10.2196/44965 PMC10022650

[32] Hwang S , Urbanowicz R , Lynch S , et al. Toward predicting 30‐Day readmission among oncology patients: identifying timely and actionable risk factors. JCO Clin Cancer Inform. 2023;7:e2200097. 36809006 10.1200/CCI.22.00097 PMC10476733

[33] Liu H , Zhang X , Liu H , Chong ST . Using machine learning to predict cognitive impairment among Middle‐Aged and older Chinese: A longitudinal study. Int J Public Health. 2023;68:1605322. 36798738 10.3389/ijph.2023.1605322 PMC9926933

[34] Basta M , John Simos N , Zioga M , et al. Personalized screening and risk profiles for mild cognitive impairment via a machine learning framework: implications for general practice. Int J Med Inform. 2023;170:104966. 36542901 10.1016/j.ijmedinf.2022.104966

[35] Joyce DW , Kormilitzin A , Smith KA , Cipriani A . Explainable artificial intelligence for mental health through transparency and interpretability for understandability. npj Digital Medicine. 2023;6(1):6. 36653524 10.1038/s41746-023-00751-9 PMC9849399

[36] van Schaik P , Peng Y , Ojelabi A , Ling J . Explainable statistical learning in public health for policy development: the case of real‐world suicide data. BMC Med Res Methodol. 2019;19(1):152. 31315579 10.1186/s12874-019-0796-7 PMC6636096

[37] Vyas A , Aisopos F , Vidal ME , Garrard P , Paliouras G . Identifying the presence and severity of dementia by applying interpretable machine learning techniques on structured clinical records. BMC Med Inform Decis Mak. 2022;22(1):271. 36253849 10.1186/s12911-022-02004-3 PMC9578246

[38] Mesripour A , Golchin S . Vitamin B6 antidepressant effects are comparable to common antidepressant drugs in bacillus‐calmette‐guerin induced depression model in mice. Iran J Psychiatry. 2022;17(2):208‐216. 36262766 10.18502/ijps.v17i2.8911 PMC9533344

[39] Akinrinde AS , Oyewole SO , Ola‐Davies OE . Supplementation with sesame oil suppresses genotoxicity, hepatotoxicity and enterotoxicity induced by sodium arsenite in rats. Lipids Health Dis. 2023;22(1):14. 36707815 10.1186/s12944-022-01760-5 PMC9881342

[40] Knapik JJ , Trone DW , Steelman RA , Farina EK , Lieberman HR . Associations between Clinically‐Diagnosed medical conditions and dietary supplement use: the US military dietary supplement use study. Public Health Nutr. 2023;26:1238‐1253. 36775272 10.1017/S1368980023000095 PMC10346078

[41] Noshiro K , Umazume T , Inubashiri M , Tamura M , Hosaka M , Watari H . Association between Edinburgh postnatal depression scale and serum levels of ketone bodies and vitamin D, thyroid function, and iron metabolism. Nutrients. 2023;15(3):768. 36771476 10.3390/nu15030768 PMC9920872

[42] Wang Y , Liu J , Compher C , Kral TVE . Associations between dietary intake, diet quality and depressive symptoms in youth: A systematic review of observational studies. Health Promot Perspect. 2022;12(3):249‐265. 36686054 10.34172/hpp.2022.32 PMC9808911

[43] Zhang T , Cui X , Zhang Y , et al. Inflammation mediated the effect of dietary fiber on depressive symptoms. Front Psychi. 2023;13:989492. 10.3389/fpsyt.2022.989492 PMC9874690 36713916

[44] Campbell RK , Tamayo‐Ortiz M , Cantoral A , et al. Maternal prenatal psychosocial stress and prepregnancy BMI associations with fetal iron status. Curr Develop Nutr. 2020;4(2):nzaa018. 10.1093/cdn/nzaa018 PMC7026381 32099952

[45] Daniels NF , Burrin C , Chan T , Fusco F . A systematic review of the impact of the first year of COVID‐19 on obesity risk factors: a pandemic fueling a pandemic? Curr Develop Nutr. 2022;6(4):nzac011. 10.1093/cdn/nzac011 PMC8989548 35415391

[46] Gonzalez‐Nahm S , Marchesoni J , Maity A , et al. Maternal Mediterranean diet adherence and its associations with maternal prenatal stressors and child growth. Curr Develop Nut. 2022;6(11):nzac146. 10.1093/cdn/nzac146 PMC9665863 36406812

[47] Hardman RJ , Meyer D , Kennedy G , Macpherson H , Scholey AB , Pipingas A . Findings of a pilot study investigating the effects of Mediterranean diet and aerobic exercise on cognition in cognitively healthy older people living independently within aged‐care facilities: the lifestyle intervention in independent living aged care (LIILAC) study. Curr Develop Nut. 2020;4(5):077. 10.1093/cdn/nzaa077 PMC7228438 32440639

[48] Hartwell ML , Khojasteh J , Wetherill MS , Croff JM , Wheeler D . Using structural equation modeling to examine the influence of social, behavioral, and nutritional variables on health outcomes based on NHANES data: addressing complex design, nonnormally distributed variables, and missing information. Curr Develop Nutr. 2019;3(5):nzz010. 10.1093/cdn/nzz010 PMC6465451 31008441

[49] Kracht CL , Swyden KJ , Weedn AE , Salvatore AL , Terry RA , Sisson SB . A structural equation modelling approach to understanding influences of maternal and family characteristics on feeding practices in young children. Curr Develop Nutr. 2018;2(9):nzy061. 10.1093/cdn/nzy061 PMC6163107 30283915

[50] Lim S , Tellez M , Ismail AI . Chronic stress and unhealthy dietary behaviors among low‐income African‐American female caregivers. Curr Develop Nut. 2020;4(3):nzaa029. 10.1093/cdn/nzaa029 PMC7085306 32215356

[51] McCusker MR , Bazinet RP , Metherel AH , et al. Nonesterified fatty acids and depression in cancer patients and caregivers. Curr Develop Nut. 2020;4(11):nzaa156. 10.1093/cdn/nzaa156 PMC7792569 33447694

[52] Huang AA , Huang SY . Increasing transparency in machine learning through bootstrap simulation and shapely additive explanations. PLoS One. 2023;18(2):e0281922. 36821544 10.1371/journal.pone.0281922 PMC9949629

[53] Keir G , Hu W , Filippi CG , Ellenbogen L , Woldenberg R . Using artificial intelligence in medical school admissions screening to decrease inter‐ and intra‐observer variability. JAMIA Open. 2023;6(1):ooad011. 36819893 10.1093/jamiaopen/ooad011 PMC9936956

