# Supplementary material for: Exploring Depression and Nutritional Covariates Amongst US Adults using Shapely Additive Explanations
Source: Health Sci Rep. 2023 Oct 20;6(10):e1635. doi: 10.1002/hsr2.1635 (PMC10588337; doi:10.1002/hsr2.1635)
Supplement: Supplementary file 1 — Supporting information. [file HSR2-6-e1635-s001.docx]

Supplemental Materials

**S1: SHAP Explanation for Total Choline (mg)**


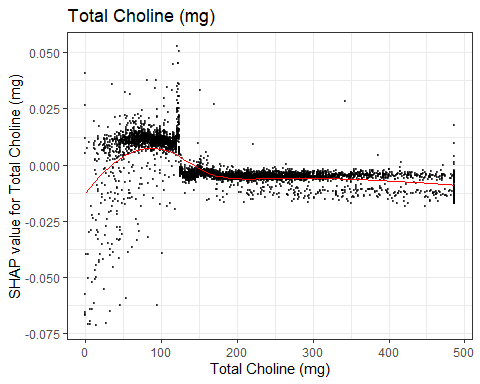


**Legend:** SHAP explanation, covariate value on the x-axis, change in log-odds on the y-axis, red line represents the relationship between the covariate and log-odds for PHQ-9 greater than or equal to 10, each black dot represents an observation.

**S2: SHAP Explanation for Caffeine (mg)**


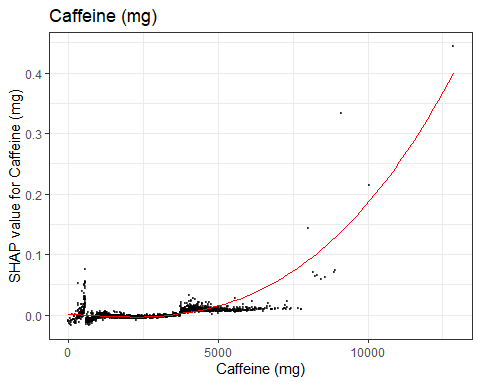


**Legend:** SHAP explanation, covariate value on the x-axis, change in log-odds on the y-axis, red line represents the relationship between the covariate and log-odds for PHQ-9 greater than or equal to 10, each black dot represents an observation.

**S3: SHAP Explanation for Low Fat and Low Cholesterol Diet**


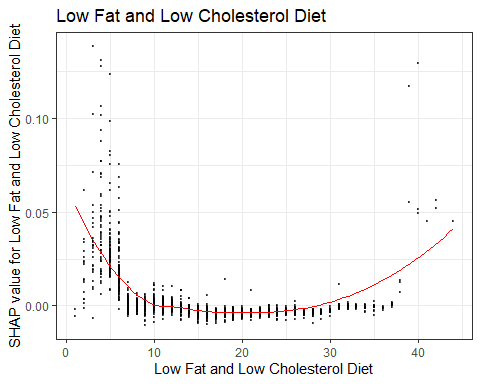


**Legend:** SHAP explanation, covariate value on the x-axis, change in log-odds on the y-axis, red line represents the relationship between the covariate and log-odds for PHQ-9 greater than or equal to 10, each black dot represents an observation.

**S4: SHAP Explanation for Alpha Tocopherol Intake (mg)**


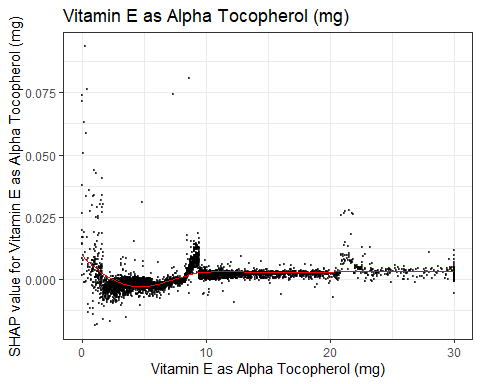


**Legend:** SHAP explanation, covariate value on the x-axis, change in log-odds on the y-axis, red line represents the relationship between the covariate and log-odds for PHQ-9 greater than or equal to 10, each black dot represents an observation.

**S5: SHAP Explanation for Vitamin K intake (mcg)**


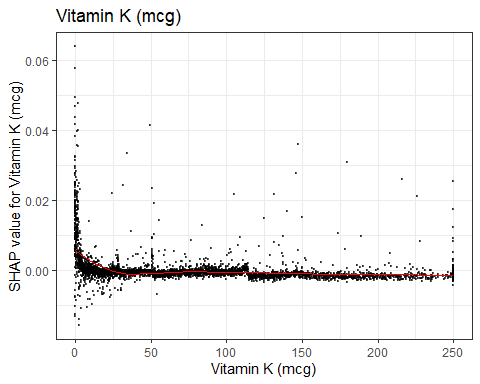


**Legend:** SHAP explanation, covariate value on the x-axis, change in log-odds on the y-axis, red line represents the relationship between the covariate and log-odds for PHQ-9 greater than or equal to 10, each black dot represents an observation.

**S6: SHAP Explanation for Grams of Fiber**


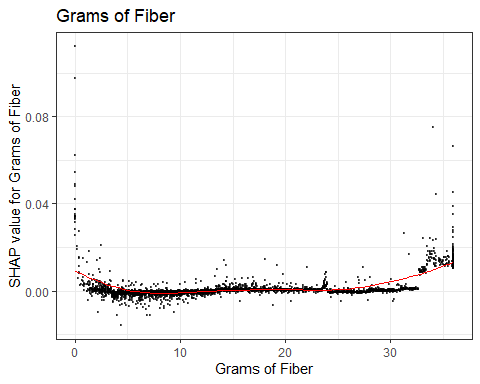


**Legend:** SHAP explanation, covariate value on the x-axis, change in log-odds on the y-axis, red line represents the relationship between the covariate and log-odds for PHQ-9 greater than or equal to 10, each black dot represents an observation.

**S7: SHAP Explanation for Phosphorus (mg)**


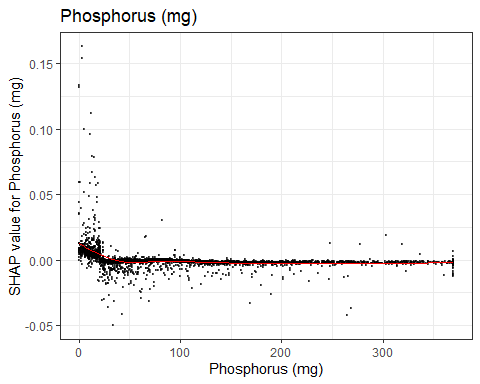


**Legend:** SHAP explanation, covariate value on the x-axis, change in log-odds on the y-axis, red line represents the relationship between the covariate and log-odds for PHQ-9 greater than or equal to 10, each black dot represents an observation.

**S8: SHAP Explanation for Grams of Protein Intake**


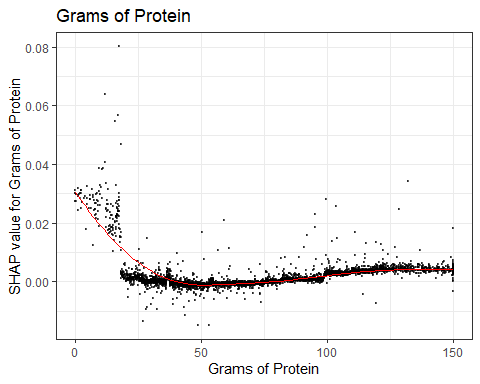


**Legend:** SHAP explanation, covariate value on the x-axis, change in log-odds on the y-axis, red line represents the relationship between the covariate and log-odds for PHQ-9 greater than or equal to 10, each black dot represents an observation.

**S9: SHAP Explanation for Lutein Zeaxanthin intake (mcg)**


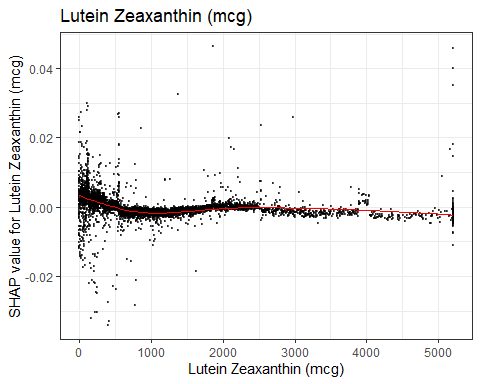


**Legend:** SHAP explanation, covariate value on the x-axis, change in log-odds on the y-axis, red line represents the relationship between the covariate and log-odds for PHQ-9 greater than or equal to 10, each black dot represents an observation.

**S10: SHAP Explanation for Folate Intake (mcg)**


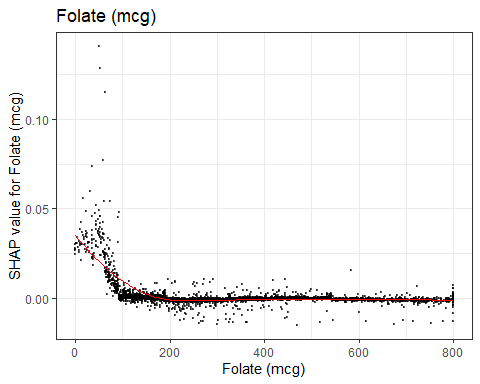


**Legend:** SHAP explanation, covariate value on the x-axis, change in log-odds on the y-axis, red line represents the relationship between the covariate and log-odds for PHQ-9 greater than or equal to 10, each black dot represents an observation.

**S11: SHAP Explanation for Iron Intake (mg)**


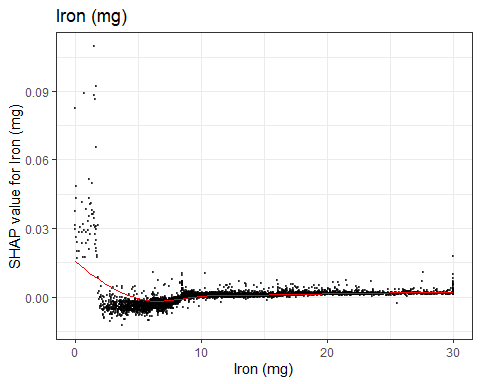


**Legend:** SHAP explanation, covariate value on the x-axis, change in log-odds on the y-axis, red line represents the relationship between the covariate and log-odds for PHQ-9 greater than or equal to 10, each black dot represents an observation.

**S12: SHAP Explanation for Sodium Intake (mg)**


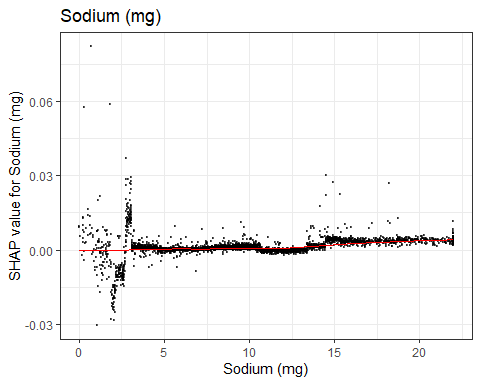


**Legend:** SHAP explanation, covariate value on the x-axis, change in log-odds on the y-axis, red line represents the relationship between the covariate and log-odds for PHQ-9 greater than or equal to 10, each black dot represents an observation.

**S13: SHAP Explanation for Theobromine Intake (mg)**


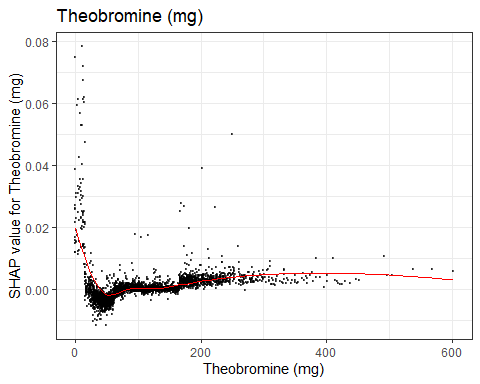


**Legend:** SHAP explanation, covariate value on the x-axis, change in log-odds on the y-axis, red line represents the relationship between the covariate and log-odds for PHQ-9 greater than or equal to 10, each black dot represents an observation.

**S14: SHAP Explanation for Copper intake (mg)**


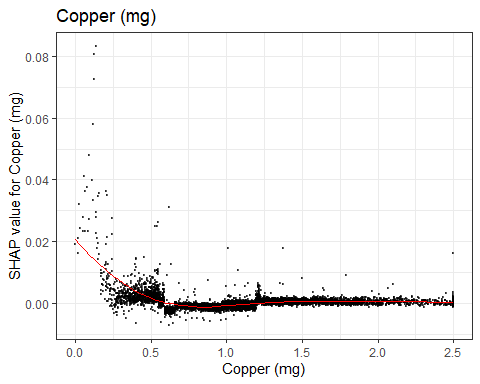


**Legend:** SHAP explanation, covariate value on the x-axis, change in log-odds on the y-axis, red line represents the relationship between the covariate and log-odds for PHQ-9 greater than or equal to 10, each black dot represents an observation.

**S15: SHAP Explanation for Magnesium Intake (mg)**


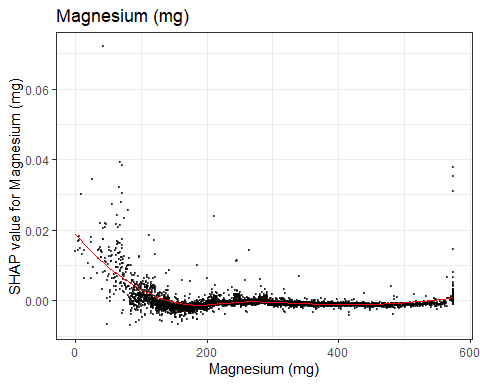


**Legend:** SHAP explanation, covariate value on the x-axis, change in log-odds on the y-axis, red line represents the relationship between the covariate and log-odds for PHQ-9 greater than or equal to 10, each black dot represents an observation.

**S16: SHAP Explanation for Food Folate Intake (mcg)**


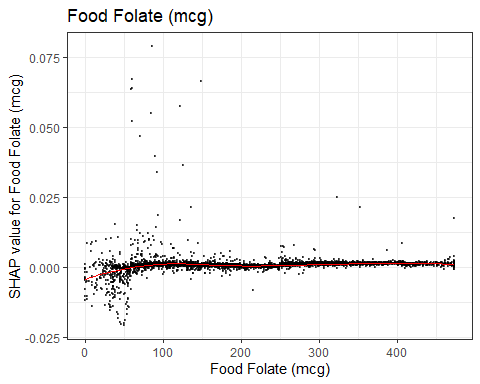


**Legend:** SHAP explanation, covariate value on the x-axis, change in log-odds on the y-axis, red line represents the relationship between the covariate and log-odds for PHQ-9 greater than or equal to 10, each black dot represents an observation.

**S17: SHAP Explanation for Vitamin B12 (mcg)**


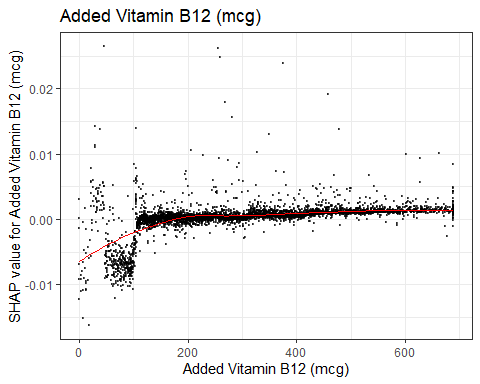


**Legend:** SHAP explanation, covariate value on the x-axis, change in log-odds on the y-axis, red line represents the relationship between the covariate and log-odds for PHQ-9 greater than or equal to 10, each black dot represents an observation.

**S18: SHAP Explanation for Zinc (mg)**


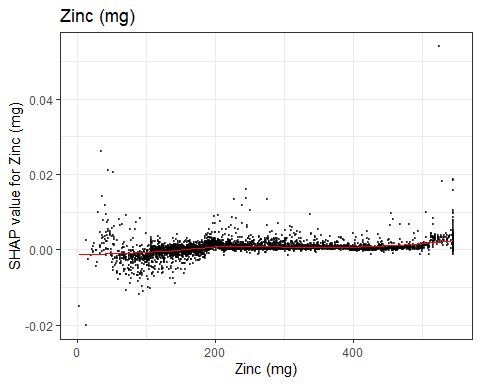


**Legend:** SHAP explanation, covariate value on the x-axis, change in log-odds on the y-axis, red line represents the relationship between the covariate and log-odds for PHQ-9 greater than or equal to 10, each black dot represents an observation.

**S19: SHAP Explanation for Total Folate (mcg)**


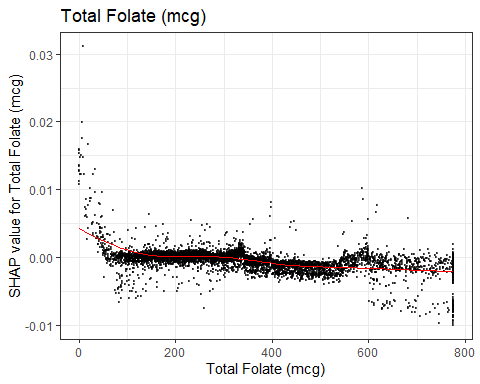


**Legend:** SHAP explanation, covariate value on the x-axis, change in log-odds on the y-axis, red line represents the relationship between the covariate and log-odds for PHQ-9 greater than or equal to 10, each black dot represents an observation.

**S20: SHAP Explanation for Folate DFE Intake (mcg)**


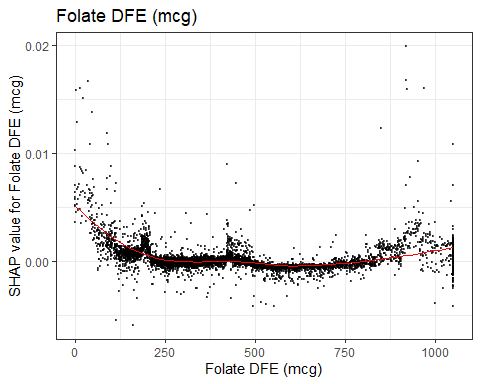


**Legend:** SHAP explanation, covariate value on the x-axis, change in log-odds on the y-axis, red line represents the relationship between the covariate and log-odds for PHQ-9 greater than or equal to 10, each black dot represents an observation.
